# Migration of African healthcare professionals: the need for equitable healthcare worker migration

**DOI:** 10.11604/pamj.2025.51.24.47894

**Published:** 2025-05-28

**Authors:** Estifanos Haimanot, Mishra Kartik, Hanif Miah

**Affiliations:** 1 University of Nis, Faculty of Medicine, Nis, Serbia,; 2 Saint James School of Medicine, Arnos Vale, Kingstown, Saint Vincent and the Grenadines

**Keywords:** Healthcare professionals, Africa, equitable, migration, health workers, brain drain

## Abstract

Healthcare professional migration has been a major issue in the African healthcare system. This is due to push factors such as economic instability, inadequate work conditions, insufficient support, and a lack of infrastructure, which have all contributed to the increased migration rates in sub-Saharan countries. On the contrary, pull factors like optimal work conditions, better work-life balance, and financial benefits have attracted African healthcare workers to settle abroad, where this issue has been exacerbated during the post-pandemic COVID-19 era. It is crucial for governments, healthcare organizations, and stakeholders to invest in proper resources, training, and autonomy to better support the working conditions and rights of African healthcare workers. Governments, Non-Governmental Organizations (NGOs), healthcare organizations and stakeholders must utilize a global agenda and implement policies, partnerships, and technological solutions for a more equitable, accessible, and dependable healthcare system.

## Essay

Globalized migration is commonly defined as a voluntary movement of workers seeking better job opportunities and employment conditions, which has significant implications for equitable health worker migration in Africa [1]. According to the World Health Organization (WHO), African countries in the Global South experience escalating conflicts due to the mass migration of health professionals to developed nations [1,2]. The “brain drain” involves skilled individuals migrating across national boundaries. Conversely, “brain gain” refers to countries benefiting from skills gained through migration, while “brain exchange” denotes the global movement of skilled workers for employment and training [1,2]. Studies indicate that about 20% of doctors in industrialized countries come from abroad, creating significant shortages in developing countries, including those in Africa, while simultaneously fueling increased migration to more developed nations [1-3].

Against this backdrop, this article delves into the migration patterns of Africa´s healthcare professionals (HCPs), investigating the underlying causes, formidable challenges, and potential opportunities for equitable health worker migration within the continent [1,2]. Moreover, it emphasizes the critical role of global health workforce planning in addressing healthcare migration, advocating for a more comprehensive global approach that considers the needs and challenges of both source and destination countries [1,2,4]. Efforts to strengthen healthcare systems, promote fair labour practices and increase investments in healthcare education and training are deemed essential for achieving equitable distribution of health workers within Africa and across the global healthcare landscape [1,2,5].

**Figure 1:**
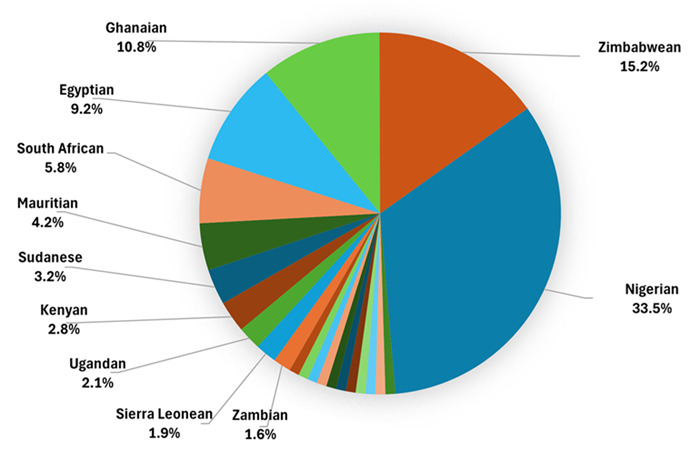
African healthcare professionals migration by national health service staff in England as of March 2021

According to recent data, there has been a significant increase in the migration of African healthcare professionals (HCPs) to developed countries in the Global North, particularly the US and England [6]. The number of African international medical graduates (IMGs) practicing in the United States in 2022 is shown in Table 1, while Figure 1 illustrates the representation of African HCPs in the NHS workforce in England as of March 2021 [6].

**Table 1:** number of international medical graduates (IMGs) in the United States (US) based on various African countries in 2022

Countries in Africa	African IMGs in the US
Sudan	419
Other (non-sub-Saharan)	643
Ethiopia	666
Ghana	746
Sub-Saharan	941
South Africa	1709
Nigeria	3669
Egypt	4791
African healthcare workforce in the US	13584 (1.4%)

The departure of healthcare professionals from various African countries exacerbates existing shortages, particularly in rural and underserved areas, where healthcare needs are the most prominent [6]. Consequently, this leads to reduced access to healthcare services for local communities, forcing patients to travel far distances or endure longer wait times for care [6]. A research study conducted in South Africa highlights how mass migration further undermines healthcare access in rural areas and facilitates a disproportionate distribution of healthcare workers [3]. For instance, socioeconomic barriers and migration patterns demonstrate that while 46.3% of healthcare workers reside in rural areas, only a small percentage of doctors (12%), general practitioners (27%), dentists (7%), psychologists (6%), and nurses (19%) specifically practice in these areas in South Africa [3]. As such, this discrepancy compromises the well-being, access, and quality of care of communities in these regions [3,4].

**Reasons for migration and opportunities:** there are various factors and opportunities as to why African healthcare workers choose to migrate. A mixed-method comparative study examined 301 items in a database, applying inclusion/exclusion criteria and synthesizing scoping reviews to identify factors promoting migration for African healthcare workers [1]. Results from literature derived from migration patterns, surveys, and critical informant interviews indicated that there is a level of uncertainty and consideration when it comes to migration decisions [1]. Push factors that drive outward migration for African workers include poor conditions, limited career growth, HIV/MDR-TB burdens, high living costs, and job insecurity [4]. Conversely, pull factors for African health worker migration include higher pay, better working conditions, career opportunities, and family security [4].

Furthermore, in healthcare, burnout is a common hazard, affecting patients, healthcare workers, and the broader healthcare system. It stems from prolonged exposure to demanding work, impacting job performance, satisfaction, and psychological health and wellness [7,8]. During the COVID-19 pandemic, approximately 38% of healthcare workers in Ghana and Kenya reported job dissatisfaction, with about 70% experiencing burnout [8]. The current challenges of the COVID-19 pandemic exacerbate the systemic problems that have plagued the underperforming African healthcare system [8]. Push factors like poor infrastructure, management, unequal resource allocation, and high patient volume hinder African healthcare system growth [8]. Addressing these push and pull factors is crucial for fostering equitable healthcare workforce migration in Africa, ensuring that healthcare professionals are motivated to stay and contribute to the improvement of healthcare services across the continent [8].

**Impact of migration on home and destination countries:** the flow of health professionals from low-income to high-income countries is a significant contributor to the further weakening of already fragile health systems. Researchers concluded that in half of the countries in sub-Saharan Africa, more than 30% of the physicians trained locally are “lost” because of migration [7]. In recent decades, high-income countries like the UK, the US, and Canada have addressed domestic shortages by recruiting health professionals internationally, leading to critical shortages in low-income countries [7]. This recruitment has sparked ethical debate, prompting the WHO to develop a voluntary code of practice on international health personnel recruitment in 2010 to protect health systems in low-income, transitioning, and small island states [7,8]. Despite efforts, migration of healthcare workers from Africa exacerbates already existing inequalities in the healthcare system, which feeds a vicious cycle of scarce resources, restricted access to care, and worsened health outcomes for various communities [7,8]. Additionally, this migration makes African nations more reliant on foreign aid or international organizations to address the resulting gaps, offering short-term respite but failing to address the underlying systemic issues driving migration or the long-term development of sustainable equitable healthcare systems [8]. On the other hand, employers from destination countries benefit from this migration by obtaining much-needed staff, potentially at lower wages, reflecting the dynamics of a “true” market economy [7,8]. However, the benefit comes with downsides, including the extra cost of international recruitment and delays in staff training for other health systems striving to reach full capacity. Moreover, complaints from patients due to language barriers further complicate the situation [8].

**Discouraging migration, how to boost healthcare:** the migration of African healthcare workers has resulted in a breakdown of healthcare systems and a substantial decrease in medical staff shortages [7]. Governments, NGOs and policymakers should offer competitive salaries and benefits to make healthcare positions more desirable in Africa [1,3,5]. African local governments can boost workforce equity by investing in local education and healthcare training, establishing more medical and nursing schools, and specialized training centers in rural regions, as well as providing career advancement opportunities to further motivate African HCPs [1,5]. Furthermore, managed migration processes could require migrating healthcare workers to serve a minimum number of years in their home country before deciding to practice abroad [5]. Destination countries can enact restrictive regulations for HCPs, while African countries can implement guidelines to discourage migration [1,5].

On a macro-level, efforts are needed to address corruption and political instability in combating vulnerabilities in the African healthcare system [3,5]. This consists of tackling corruption among sub-Saharan governments in Africa, reducing bureaucracy, and ensuring effective health workforce planning [3,5,9]. Ensuring adequate access to essential medical equipment is key to also training more HCPs and increasing production in both rural/urban communities within sub-Saharan Africa [3,9,10]. This would consist of capacity building, research opportunities, knowledge sharing, training, mentorship, and networking, which can enable HCPs to benefit from global expertise locally [9,10]. Governments and NGOs can promote global-local collaboration among HCPs through exchange programs, interactive partnerships, and twinning programs, which would foster expertise sharing [9,10].

At last, technology can have a significant role in decreasing healthcare migration in Africa. For one, telemedicine can improve healthcare access in remote areas by offering remote consultations, reducing the need for physical relocation, and waiting times [3,10]. Moreover, technology can help enable online training and e-learning platforms for HCPs, expanding educational resources and professional development opportunities. This helps African HCPs access quality education and care, reducing migration [10]. Lastly, AI and data analytics offer promising tools for healthcare post-COVID-19. These tools can help HCPs analyze large data sets, identify trends, and conduct informed decision-making for predicting health outcomes, optimizing treatments, and diagnosing diseases. By leveraging artificial intelligence to patient-related healthcare outcomes, HCPs can provide high-quality care locally, reducing the need for migration [10].

## Conclusion

Overall, globalization has increased the shortage of frontline HCPs due to the mass migration of African professionals. The African healthcare system has significantly deteriorated over the years, facing numerous systemic issues. Reforming Africa's healthcare system is essential to mitigate rising migration rates and bolster its foundational values, ensuring improved accessibility, quality, and sustainability of healthcare services. A global strategy that takes into account the requirements and difficulties of both source and destination countries is needed to address healthcare migration. Achieving equitable health worker migration necessitates collaborative efforts among governments, healthcare organizations, and stakeholders to improve the healthcare system, boost funding for healthcare education and training, and support ethical labor practices. By doing so, we not only bolster the healthcare workforce in Africa but also contribute to a more equitable global healthcare system, where all individuals, regardless of geography, have access to quality care.
